# Comparative Mitogenomic Analysis of Three *Chionea* Species (*Tipulomorpha*: *Limoniidae*): Insights into Phylogenetic Relationships and Selection Pressure

**DOI:** 10.3390/insects16070720

**Published:** 2025-07-14

**Authors:** Yufeng Feng, Wei Cen, Kenneth B. Storey, Lingjuan Liu, Danna Yu, Jiayong Zhang

**Affiliations:** 1 College of Life Sciences, Zhejiang Normal University, Jinhua 321004, China; 2 Department of Biology, Carleton University, Ottawa, ON K1S5B6, Canada; 3 Longquan Conservation Center of Qianjiangyuan-Baishanzu National Park, Longquan 323000, China; 4 Key Lab of Wildlife Biotechnology, Conservation and Utilization of Zhejiang Province, Zhejiang Normal University, Jinhua 321004, China

**Keywords:** *Chionea*, mitochondrial genome, phylogenetic relationship, selective stress analysis, *crane fly*

## Abstract

*Chionea* is classified within the Tipuloidea superfamily and predominantly inhabits cold regions. However, its phylogenetic relationships remain contentious. In this study, the first three mitogenomes of *Chionea* (Diptera: Limoniidae) sampled in northeastern China (Jilin region) were sequenced, and their phylogenetic relationships were reconstructed by integrating these sequences with 30 additional Tipuloidea mitogenomes retrieved from NCBI. Unlike other Tipuloidea species, which are predominantly distributed in relatively warmer regions, this research investigates whether positive selection has acted on the mitogenomes of these three *Chionea* species due to environmental pressures, thereby elucidating key evolutionary drivers for *Chionea*. The three mitogenomes of *Chionea* exhibit characteristic features typical of insect mitogenomes, comprising 13 protein-coding genes (PCGs), 2 ribosomal RNA genes (16S rRNA and 12S rRNA), 22 transfer RNA genes (tRNA), and a single non-coding control region (D-loop). Notably, the secondary structure of trnS1 lacks the DHU arm in all three samples, and UUA (Leu) emerges as the most frequently utilized codon. Furthermore, the *COX2* and *ND5* genes utilize incomplete stop codons “T”. Utilizing these 13 PCGs, we reconstructed the internal phylogenetic relationships within Tipuloidea, revealing that *Chionea tianhuashana* and *C. sphaerae* form sister branches, while (*C. tianhuashana* + *C. sphaerae*) constitutes a sister branch to *C. crassipes*. Moreover, our analysis confirms the monophyly of Tipulidae, *Tipula*, and *Nephrotoma* as well as the polyphyly of Tipulinae, Chioneinae, and Limoniidae. In the branch site model analysis, three positively selected sites were detected when *Chionea* was designated as the foreground branches: *COX3* (at position 242), *ND5* (at position 535), and *ND6* (at position 138).

## Simple Summary

*Chionea* is a genus within the Tipuloidea superfamily that exhibits a broad distribution across cold regions. Due to incomplete molecular data, the phylogenetic relationships of *Chionea* have remained contentious. As an effective molecular marker, mitochondrial genomes (mitogenomes) can be utilized to help elucidate the phylogenetic relationships of *Chionea*. Unlike other species within Tipuloidea, *Chionea* has adapted to long-term survival in low-temperature environments. Adaptation to such conditions may require increased energy expenditure, while mitochondria function as the primary centers for energy metabolism, providing the majority of ATP required for physiological and biochemical processes through oxidative phosphorylation. Consequently, the mitogenome was analyzed to detect positive selection in *Chionea*’s PCGs.

## 1. Introduction

Nematocera is classified within the order Diptera, which encompasses both Tipulomorpha and Culicomorpha. These groups play crucial roles in ecosystems, with species richness serving as a significant indicator of habitat quality [1]. As of May 2025, NCBI has documented 13,912 mitochondrial gene entries for Tipulomorpha; however, only 28 represent complete or near-complete mitogenomes. Tipulomorpha larvae occupy varied habitats such as aquatic systems, wetlands, humid substrates, and decomposing timber. Unlike their larvae, adult Tipulomorpha predominantly inhabit moist temperate zones, commonly found among riparian vegetation adjacent to forest water bodies worldwide [2]. The superfamily Tipuloidea, which falls under Diptera, comprises four families: Tipulidae, Pediciidae, Limoniidae and Cylindrotomidae. These families exhibit a global distribution and encompass over 500 genera and subgenera, along with more than 15,000 species [1,3].Within the order Diptera lies one of the most active insect groups during winter months, with certain species having adapted to thrive in colder climates. For instance, the genus *Chionea* comprises 14 species in the Paleoarctic and 18 species in the Nearctic, demonstrating a pan-Arctic distribution pattern [4].

Mitochondria are essential organelles responsible for aerobic respiration and play a critical role in energy production as well as the maintenance of normal cellular functions. Compared to Protozoa, the mitochondrial structure in Metazoa demonstrates a higher degree of complexity and distinct morphological features. In animals, mitochondrial DNA exists as a double-stranded circular chromosome, typically containing 37 evolutionarily conserved genes: 13 protein-coding genes (PCGs), 22 transfer RNA (tRNA) genes, and 2 ribosomal RNA (rRNA) genes. These genes encode key components of the respiratory chain and ATP synthase complexes, whose expression levels may be regulated under varying environmental conditions, thereby enhancing the organism’s adaptability to environmental changes [5]. Mitochondrial genomes have become a cornerstone tool in molecular systematics and evolutionary biology, owing to their structural stability and evolutionary conservation across taxa. These characteristics make them particularly valuable for species delineation, phylogenetic reconstruction, and evolutionary mechanism studies [6,7,8,9,10,11,12]. Transfer RNA plays a critical role in transporting amino acids to the ribosomes located within the mitochondria and is indispensable for the protein synthesis process occurring within these organelles [13]. Mitochondrial DNA encompasses 13 essential protein-coding genes (PCGs) that constitute the core components of oxidative phosphorylation—the primary process by which cells generate energy. These genes encode critical subunits of the electron transport chain, which function in a coordinated manner to synthesize ATP, the principal energy currency driving virtually all cellular activities [14]. It is evident that research on mitogenomes in invertebrates remains relatively limited compared to that in vertebrates [15]. A breakthrough occurred in 1985 when Clary et al. first sequenced the entire mitochondrial genome of *Drosophila yakuba*, advancing molecular entomology and establishing the first reference mitogenome for insect phylogenetic studies [16]. With representatives across 11 taxonomic orders and comprising >50% of documented animal biodiversity, insects presently constitute the most evolutionarily successful metazoan lineage [17]. The length of insect mitogenomes typically varies between 14 and 20 kb [18]. Mitochondrial DNA exhibits low recombination rates, high gene conservation, rapid evolutionary rates, and strict maternal inheritance, making it a vital molecular marker for investigating species origin and evolution [19,20]. Furthermore, advancements in sequencing technology have significantly enhanced the cost-effectiveness and efficiency of mitochondrial DNA acquisition. As a transformative technological innovation, next-generation sequencing provides indispensable technical support for indispensable systematic position and phylogenetic relationships.

With advancements in molecular technology, the phylogenetic relationships among biological systems are becoming increasingly well-defined. However, the systematic phylogenetic relationships within Tipuloidea remain relatively unresolved. Kang et al. described previous results of phylogenetic analyses of Tipuloidea as “entirely unsatisfactory” [21]. Building on the morphological differences between ‘long-palped’ species in Tipulidae sensu stricto and ‘short-palped’ species in Limoniidae, Ribeiro et al. carried out a qualitative analysis of the group’s characteristics and presented the first formal taxonomic definition [22]. Alexander et al. and Savchenko et al. proposed the first evolutionary hypothesis for Tipuloidea based on certain ambiguously defined relationships among the taxa [23,24]. Starý performed an analysis of the morphological characteristics of adult insects to expound the phylogenetic position of this family and its related families, proposing the separation of Pediciinae from Limoniidae to establish a distinct family [25]. However, he did not provide clear definitions for the classification of each subfamily, a limitation that was subsequently questioned by Song et al. [26]. Kania et al. utilized 41 specimens to reconstruct the monophyletic Limoniinae (including Pediciinae) as the sister to (Cylindrotominae + Tipulinae) [27]. Kolcsár et al. investigated the phylogenetic relationships within Tipuloidea (comprising Cylindrotomidae, Limoniidae, and Tipulidae) based on larval and pupal characteristics and redefined the taxonomic status of Tipuloidea and several associated subfamilies [28]. Ribeiro performed a comprehensive phylogenetic analysis of Tipulomorpha, with a particular focus on Limnophilinae (Limoniidae). He proposed that Limoniidae is the sister group to (Cylindrotomidae and Tipulidae), while Limnophilinae, Limoniinae, and Chioneinae are considered paraphyletic [29]. Lukashevich and Ribeiro elucidated the monophyly of the Tipulidae family, including *Tipunia*, through their comprehensive analysis of Tipulomorpha fossils [30]. Furthermore, Kang et al. utilized whole transcriptome data to reassess the taxonomic status of several species within Tipuloidea, supporting the phylogenetic relationship of (Pediciidae + (Limoniidae + (Cylindrotomidae + Tipulidae))) [31]. Through Bayesian phylogenetic reconstruction, Zhang et al. demonstrated strong statistical support for the sister-taxon relationship between Trichoceridae and Tipuloidea, advancing our understanding of dipteran phylogeny. These findings do not corroborate the hypothesis that Tipulomorpha represents the earliest branch within Diptera. Additionally, their reconstructed phylogenetic tree suggests that the Limoniidae family is paraphyletic [32]. Challenging traditional classifications, Petersen et al.’s rigorous phylogenetic reconstruction rejected the monophyletic status of Limoniidae, instead supporting hypotheses of polyphyly/paraphyly based on multiple gene concordance analyses [23].

The principle of “survival of the fittest” represents a fundamental mechanism in natural selection. The species that persist are not necessarily the most physically robust, but rather those that demonstrate the greatest adaptability to changing environmental conditions [33]. The survival, persistence, and evolution of organisms primarily depend on their adaptive evolution in response to environmental changes, which is predominantly manifested through physiological and biochemical characteristics within the organism, as well as adjustments in external morphology and behavioral patterns to accommodate varying environments [34,35,36]. While mitochondrial DNA (mtDNA) mutations have traditionally been viewed as evolving under a constant mutation rate according to neutral evolution theory, accumulating evidence challenges this paradigm. Contemporary genomic studies increasingly demonstrate that mitochondrial evolution may be governed by similar selective pressures and evolutionary dynamics as observed in nuclear genomes [37]. The study by Xu et al. indicates that the selective status of the mitochondrial genome (including positive selection, negative selection, and neutral selection) is closely linked to the environmental context in which organisms exist [38]. Under environmental pressure, mitochondrial genes may exhibit selective expression to facilitate adaptation to the environment and enhance survival capacity [39]. Maximum likelihood methods were employed to analyze patterns of adaptive evolution in the mitochondrial genome, demonstrating that approximately 26% (95% CI: 5.7–45%) of nonsynonymous substitutions showed evidence of positive selection. These results indicate a substantial contribution of adaptive evolution to genetic variation within mitochondrial genomes [40]. Through population genomic analyses of low- and high-altitude *Equus caballus*, Ning et al. observed an altitude-dependent decrease in genetic diversity, accompanied by signatures of positive selection in the ND6-encoded protein. These results underscore the crucial influence of hypoxic conditions on mitochondrial genome evolution in *Equus caballus* [41]. Yang et al. investigated 13 PCGs in insects, revealing that species with higher energy demands experience stronger selective pressure. Furthermore, insects exhibiting indirect flight possess a greater number of positively selected genes and are subjected to more pronounced positive selection compared to those engaging in direct flight [42].

Given mitochondria’s dual roles in cellular energetics and evolutionary adaptation, this study systematically examines mitogenomic evolution in *Chionea*, while concurrently reconstructing phylogenetic relationships within Tipulomorpha to elucidate both molecular adaptation and taxonomic diversification. We sequenced three mitochondrial genomes from *Chionea* species collected in Jilin City, China, where the annual average temperature is 13.3 °C. Furthermore, we integrated the 33 mitogenomes retrieved from NCBI with the newly sequenced mitogenomes to address the following objectives: (1) to analyze the mitochondrial genome characteristics of *Chionea*; (2) to reconstruct the phylogenetic relationships of *Chionea* within Tipulomorpha; (3) to evaluate whether the *Chionea* mitogenomes collected from Jilin are subject to positive selection during their adaptation to low-temperature environments. This study contributes new mitogenomic data resources while advancing our mechanistic understanding of cold-adaptation strategies in *Chionea*, simultaneously clarifying its phylogenetic position and evolutionary trajectory within Tipulomorpha.

## 2. Materials and Methods

### 2.1. Sample Collection and Preservation

This research utilized samples collected within Jilin City, Jilin Province, China (43°49′18′ N, 125°31′58′ E). Three samples were photographed under a Nikon SMZ-1500 zoom stereomicroscope. Detailed imaging of the mouthparts, antennae, thorax, abdomen, and legs was captured using a TSView8 camera. The optical morphological structures were subsequently measured and analyzed utilizing Adobe Illustrator CS4 software [43]. Based on their morphological characteristics, the samples were preliminarily identified as *Chionea crassipes*, *C. sphaerae* and *C. tianhuashana*. Following the completion of morphological identification, all specimens underwent secondary ethanol immersion (95%) prior to long-term cryopreservation at −80 °C in ultralow-temperature freezers.

### 2.2. DNA Isolation, PCR Amplification, and Sanger Sequencing

Following genomic DNA extraction, fragments of the COI gene were amplified using the universal insect primers LCO1490/HCO2198 [44]. Species identification was subsequently performed through sequence comparison with the NCBI BLAST database (https://blast.ncbi.nlm.nih.gov/Blast.cgi, accessed on 2 June 2024). All mitogenomic DNA was extracted from muscle tissue using Sangon’s Ezup Column Kit (Shanghai, China). For mitochondrial gene amplification, 13 overlapping primer pairs designed by Zhang et al. were employed in polymerase chain reactions (PCRs) [45]. All amplification primers were computationally designed using Primer Premier v5.0 and subsequently evaluated for specificity through BLAST analysis against the NCBI nucleotide database prior to experimental use [46]. PCR amplicons were verified through gel electrophoresis and bidirectional sequencing (Sangon Biotech, Shanghai, China). The complete mitochondrial genomes of *Chionea crassipes* (PV185784), *C. sphaerae* (PV185785), and *C. tianhuashana* (PV185786) were annotated and deposited in GenBank.

### 2.3. Gene Annotation and Sequence Analysis

Upon receiving the sequence data, sequence assembly and manual correction were performed with DNASTAR Package v7.1 (Burland TG, Totowa, NJ, USA), including base calling verification and contig assembly [47], ultimately obtaining the complete mitochondrial genome. The tRNA genes were annotated using the MITOS web server (http://mitos.bioinf.uni-leipzig.de/index.py, accessed on 15 July 2024) [48]. We retrieved the complete genome sequence of *Chionea crassipes gracilistyla* (MK941181) from NCBI (accessed on 17 July 2024) as a reference for comparative analysis. Amino acid sequences of two rRNAs and 13 PCGs were aligned via Clustal W and Mega v7.0 [49]. Visualization of the complete mitogenomes was achieved through the CGView Server (version 1.0, Grant JR et al.), with the circular map generated using default parameters (accessed on 18 March 2025) [50]. The cloverleaf secondary structures of all tRNA genes were predicted using tRNAscan-SE version 1.21 software [51] (https://lowelab.ucsc.edu/tRNAscan-SE/, accessed on 18 July 2024). Furthermore, comprehensive sequence analyses were performed with PhyloSuite (version 1.2.2), including the following: (1) nucleotide composition assessment, (2) AT- and GC-skew calculations, (3) codon usage pattern evaluation, and (4) relative synonymous codon usage (RSCU) determination [52]. AT and GC biases were calculated using the following: AT bias = (A − T)/(A + T), GC bias = (G − C)/(G + C) [53].

### 2.4. Calculation of Genetic Distance

Pairwise genetic distances among three mitochondrial genomes were determined with Mega 11.0 [54].To evaluate intra-subfamily genetic divergence, we incorporated the published mitogenome of *Symplecta hybrida* (NC030519) [55,56] from NCBI’s database. Pairwise genetic distances among the four Chioneinae species were then computed.

### 2.5. Phylogenetic Analyses

By integrating three newly sequenced mitogenomes with 30 previously published mitogenomes of Tipuloidae (Table 1), we conducted a phylogenetic analysis within this family, including samples from Cylindrotomidae (1 species), Limoniidae (13 species), Pediciidae (1 species), and Tipulidae (18 species) [28,57,58]. Certain species, such as *Conosia irrorata* and *Chionea crassipes gracilistyla*, were excluded from the phylogenetic analysis due to long-branch attraction effects and significant heterogeneity observed in the heterogeneity test [59]. Additionally, a comparison of *COX1* sequences revealed that the *Chionea crassipes gracilistyla* deposited in NCBI is 99% similar to *Neoneuromus orientalis,* suggesting it likely belongs to Megaloptera. Consequently, it was not included in the scope of phylogenetic analysis. For outgroup selection, we retrieved data for three Trichoceridae species (MW263048, NC016173, and NC016169) from the NCBI database to conduct phylogenetic analysis [60]. The PCG123 dataset was aligned with MAFFT version 7, followed by conserved region identification with Gblocks v0.91b for the PCG123 dataset [61,62]. Subsequently, the concatenated sequences were aligned using PhyloSuite v.1.2.2 and further analyzed with Geneious v.8.1.6 [52,63]. The nucleotide dataset was categorized into two groups: the PCG123 dataset and the PCG12 dataset. Using the nucleotide sequence dataset derived from 13 PCGs, we assessed substitution saturation using DAMBE v.4.2 [64]. Saturation tests demonstrated that third codon sites remained below saturation thresholds. Therefore, we employed the PCG123 dataset for phylogenetic analyses. By default, AliGROOVE was utilized to evaluate nucleotide sequence heterogeneity [64]. Evolutionary analyses (BI and ML) on the 13 PCGs dataset were performed on the concatenated PCG dataset using PartitionFinder v2.2.1 for model selection and partitioning optimization [65]. Analysis of the PCG123 dataset revealed seven distinct evolutionary partitions, as detailed in Table S1. Subsequent phylogenetic analysis was performed using the GTR + I + G model. The BI analysis employed MrBayes version 3.2 with these parameters: chain length = 10 million generations, sampling frequency = 1000 generations, convergence criterion = average SD of split frequencies ≤ 0.01 [66]. The ML analysis was implemented using RaxML v.8.2 and involved rapid inference evaluation at each node across 1000 fast replications [67]. Additionally, Tracer v.1.7.1 and FigTree v.1.4.0 were employed to assess convergence of chain stability distribution and visualize generated tree diagrams [68].

### 2.6. Selection Analysis

This study utilized the EasyCodeML program to analyze selective pressure acting on the mitochondrial genome [69]. The dN/dS ratio (ω) was computed for each PCG to evaluate selection pressures. Evolutionary pressures were classified based on ω: negative selection (ω < 1), neutral evolution (ω ≈ 1), or positive selection (ω > 1) affecting the ancestral node [70]. In the branch-site model analysis, *Chionea* was specified as the foreground branch while other Tipuloidea species served as background branches to test for positive selection signals specific to this genus. Three distinct evolutionary models were employed to examine mitogenome adaptation: (1) branch models testing lineage-specific rate variation; (2) branch-site models detecting selection at particular codons along specified branches; and (3) evolutionary branch models validating multi-branch patterns. Model comparisons included M0 (one-ratio) versus two-ratio analyses to contrast foreground (*Chionea*) and background (other species in Tipuloidea) evolutionary trajectories [71]. Finally, to understand how positive selection affects specific sites within the foreground branch, we tested Model A vs. Model A null within the branch site framework. Likelihood ratio tests (LRT) assessed model fit, while Bayesian empirical Bayes (BEB) analysis computed posterior probabilities for sites under positive selection.

## 3. Results

### 3.1. Gene Structure of Three Mitochondrial Genomes

**Figure 1:**
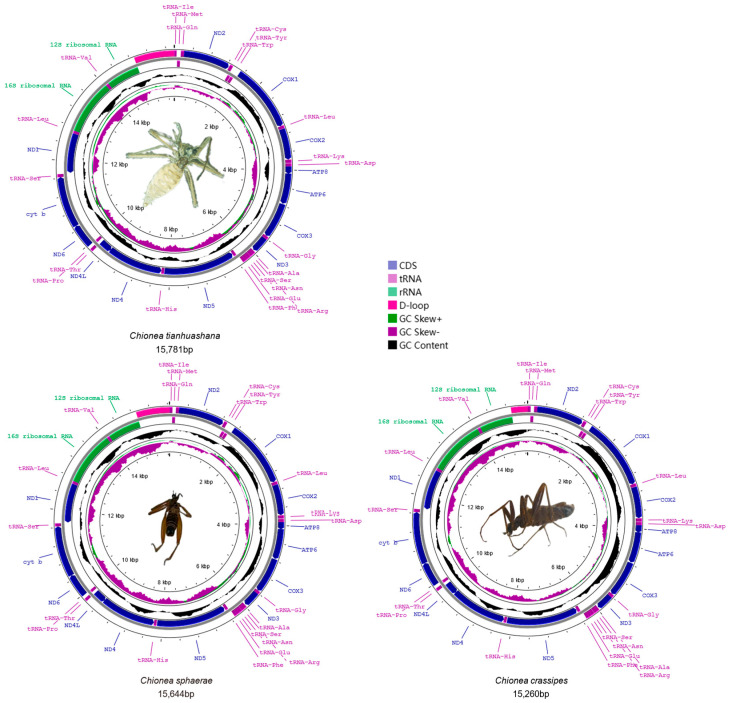
Mitogenome map of *Chionea tianhuashana*, *Chionea sphaerae* and *Chionea crassipes*. The outermost ring displays features encoded by the positive strand, while the adjacent ring corresponds to the negative strand. tRNAs are annotated using standard abbreviations. The third ring visualizes GC content, and the innermost ring (violet and green) indicates GC skew.

This study successfully obtained three nearly complete mitogenomes, excluding a part of the control region (CR). The partial mitochondrial genome of *C. tianhuashana* measured 15,781 bp in length, that of *C. sphaerae* was 15,664 bp, and that of *C. crassipes* was 15,260 bp (Figure 1). The mitogenomes of *C. tianhuashana*, *C. sphaerae* and *C. crassipes* exhibited double-stranded circular structures and comprised a total of 37 complete gene sets, including 2 rRNAs, 22 tRNAs, and 13 PCGs. Among these 37 genes, 23 were situated on the “H” chain, comprising 14 tRNAs and 9 PCGs, while the remaining 14 were located on the “L” chain with a composition of 2 rRNAs, 8 tRNAs, and 4 PCGs. The lengths of 13 PCGs for *C. tianhuashana*, *C. sphaerae* and *C. crassipes* were recorded as follows: 11,165 bp for *C. tianhuashana*, 11,159 bp for *C. sphaerae* and 11,254 bp for *C. crassipes*. Bioinformatic analysis revealed that all 13 PCGs in the three novel mitogenomes employed conventional ATN initiation codons (ATA, ATG, ATC, or ATT). Regarding stop codons, nine positions in *C. tianhuashana*, *C. sphaerae* and *C. crassipes* employed TAA as their stop codon. In contrast, two PCGs (*Cytb* and *ND1*) used TAG as their stop codon, and sequence analysis revealed the presence of incomplete stop codons (T) in both *COX2* and *ND5* genes (Table S2).

The study compared GC skew, AT skew, and A + T content across different genomic regions (whole genome, protein-coding genes, tRNAs, and rRNAs) in *C. tianhuashana*, *C. sphaerae* and *C. crassipes*. These metrics reveal biases in DNA base distribution and structural or functional constraints. The nucleotide composition of the *C. tianhuashana* genome is as follows: A = 31.2%, T = 45%, C = 11.3%, and G = 12.5%, which closely resembles that of *C. sphaerae* (A = 31.3%, T = 44.6%, C = 11.6%, and G = 12.6%) and *C. crassipes* (A = 31.1%, T = 44.1%, C = 12%, and G = 12.7%). The A + T content across the whole genomes of *C. tianhuashana*, *C. sphaerae* and *C. crassipes* is notably high at 77.2%, 77.2%, and 76.7%, respectively. Simultaneously, consistent with strand asymmetry patterns, the heavy strand exhibited negative GC skewness and positive AT skewness. Furthermore, we noted that the AT skew (+) for PCGs in all three samples exceeded that of PCGs (−), with GC skew (+) consistently being negative while GC skew (−) uniformly positive.

### 3.2. Utilization of Codons

**Figure 2:**
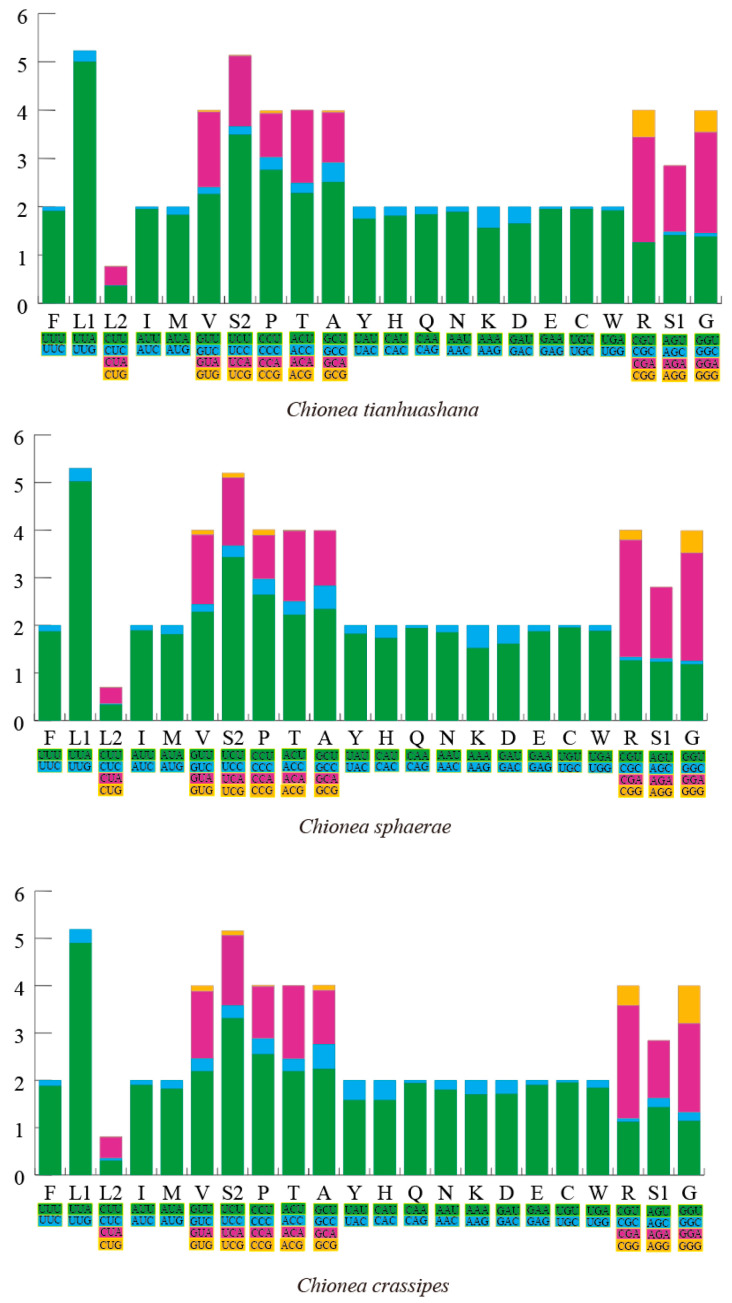
The RSCU of *C. tianhuashana*, *C. sphaerae* and *C. crassipes*. The *X*-axis shows all possible codon combinations, including synonymous codons, while the *Y*-axis indicates the corresponding RSCU values, with each codon represented by a distinct color.

The amino acid sequences of the 13 PCGs from *C. tianhuashana*, *C. sphaerae* and *C. crassipes* were successfully obtained. In these three newly sequenced mitogenomes, the overall codon usage pattern is highly comparable, with Leu1, Ile, Phe, Met, and Gly identified as the five most commonly encoded amino acids. Figure 2 illustrates the evaluation of RSCU for *C. tianhuashana*, *C. sphaerae* and *C. crassipes* in this study. *C. tianhuashana*, *C. sphaerae* and *C. crassipes* contained 3760, 3758, and 3754 codons (excluding stops), respectively. Among the 62 codons in C. *tianhuashana* and *C. sphaerae*, 28 codons exhibit higher usage frequency (RSCU > 1), whereas 34 codons demonstrate lower usage frequency (RSCU < 1). In contrast, *C. crassipes* had 29 high-frequency and 33 low-frequency codons (Table S3). The PCGs of the three mitogenomes revealed that the highest-frequency codons were UUA (Leu), AUU (Ile), and UUU (Phe), each appearing at least 325 times. The calculated RSCU values indicated that UUA (Leu) had the highest frequency of usage across all three datasets, with respective RSCU values of 5.00 for *C. tianhuashana*, 5.02 for *C. sphaerae* and 4.90 for *C. crassipes*. Conversely, the utilization frequency of codons featuring G or C as their third base was markedly low. Notably, AGG (Ser) was nearly undetectable among the three samples analyzed herein. These findings suggest a high degree of conservation in RSCU across the mitogenomes examined.

### 3.3. Transfer RNA (tRNA) and Ribosomal RNA (rRNA)

The mitogenomes of *C. tianhuashana*, *C. sphaerae* and *C. crassipes* samples all contain two rRNAs and 22 tRNAs. In the newly sequenced mitogenomes, a total of 22 structurally similar tRNAs were identified, with lengths measuring 1472 bp (*C. tianhuashana*), 1486 bp (*C. sphaerae*), and 1487 bp (*C. crassipes*). Except for the tRNA genes trnV, trnP, trnL, trnC, trnY, trnQ, trnF, and trnH, all other tRNA genes are located on the heavy (H) chain. The A + T content is higher than that of G + C across all three newly sequenced genomes. On the heavy (H) chain, the A content exceeds that of T in *C. tianhuashana*, *C. sphaerae* and *C. crassipes*. Conversely, on the light (L) strand, the A content is lower than that of T. The G content surpasses C content in both strands. The 22 tRNA molecules from these samples display typical cloverleaf-like secondary structures, with their lengths varying between 64 and 73 base pairs.

Among the tRNA gene sets of these mitochondrial genomes, excluding trnS1, the secondary structures for all remaining tRNAs are identical and conform to a standard cloverleaf model. However, trnS1 within these samples lacks the DHU arm, preventing it from forming a complete cloverleaf structure. Notable mismatches, including U-U, G-U and U-C pairs were detected in fourteen specific tRNAs (trnV, trnS2, trnP, trnT, trnH, trnF, trnR, trnA, trnL2, trnY, trnC, trnW, trnQ and trnI).

The lengths of the 12S rRNA are 786 bp (*C. tianhuashana*), 787 bp (*C. sphaerae*), and 785 bp (*C. crassipes*), while the lengths of the 16S rRNA are 1314 bp (*C. tianhuashana*), 1314 bp (*C. sphaerae*), and 1315 bp (*C. crassipes*). The A + T content for both rRNA genes across *C. tianhuashana*, *C. sphaerae* and *C. crassipes* is recorded at 80.6%, 80.9%, and 80.6%, respectively. Their GC skews exceed zero while their AT skews remain below zero. These findings indicate that AT bases are utilized more frequently than GC bases within *C. tianhuashana*, *C. sphaerae* and *C. crassipes*, with A being more prevalent than T and G being more abundant than C.

### 3.4. Genetic Distances

The genetic distance was calculated based on the *COX1* gene, and the results are summarized as follows. The genetic distance between *S*. *hybrida* and *C. tianhuashana* was measured at 15.3%, while the distances to *C. sphaerae* and *C. crassipes* were both recorded as 15.4%. The genetic distance between *C. tianhuashana* and *C. sphaerae* was determined to be 5%, and the genetic distance between *C. tianhuashana* and *C. crassipes* was 5.8%. Furthermore, the genetic distance between *C. sphaerae* and *C. crassipes* was found to be 6.1%. Results derived from *COX1* gene analysis of the three samples indicated that all inter-sample genetic distances were ≥5.5%, suggesting that *C. tianhuashana*, *C. sphaerae* and *C. crassipes* represent three distinct species (Table 2).

### 3.5. Phylogenetic Relationship

**Figure 3:**
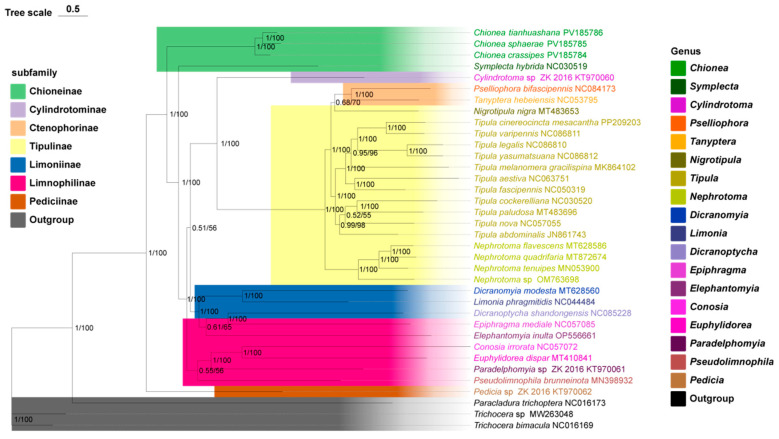
ML and BI trees for all Tipulomorpha were constructed using 13 PCGs. Different subfamilies and genera are color-coded, while the three outgroup species are grouped under a single color. The GenBank accession numbers for each species are provided following their respective names.

BI and ML revealed highly similar topologies (Figure 3) BI topology exhibited a higher confidence level and thus served as the primary reference for the phylogenetic analysis conducted in this study. At the genus level, this research reconstructed the monophyletic of *Tipula* and *Nephrotoma*. *C. tianhuashana* and *C. sphaerae* formed sister branches, while (*C. tianhuashana* + *C. sphaerae*) was identified as a sister branch to *C. crassipes*. The Tipulinae and Ctenophorinae formed sister groups. (Tipulinae + Ctenophorinae) forms a sister group with Cylindrotominae. ((Tipulinae + Ctenulicinae) + Cylindrotominae) was related to Limoniinae as a sister group. (((Tipulinae + Ctenophorinae) + Cylindrotominae) + Limoniinae) formed the sister taxa of Limnophilinae, and ((((Tipulinae + Ctenophorinae) + Cylindrotominae) + Limoniinae) + Limnophilinae) formed the sister taxa of Limnophilinae, and (((((Tipulinae + Ctenophorinae) + Cylindrotominae) + Limoniinae) + Limnophilinae) + Limnophilinae) formed the sister group of Pediciinae. At the subfamily level, Tipulinae, Limnophilinae, Limoniinae, and Chioneinae were paraphyletic, whereas Ctenophorinae was recognized as monophyletic. Given that only one sample from each of Pediciinae and Cylindrotominae was included in this phylogenetic analysis, their monophyly could not be confirmed. At the family level, Tipulidae exhibits monophyly, while Limoniidae was classified as paraphyletic. Similarly, due to having only one sample each in constructing their respective phylogenetic trees, Cylindrotomidae and Pediciidae also lack confirmation of their monophyly.

### 3.6. Positive Selection

This study utilized Bayesian inference (BI) trees with enhanced confidence levels to designate the three species of *Chionea* as foreground branches, while the remaining 30 species were assigned as background branches. A selective pressure analysis was conducted on PCGs across 33 species within Tipuloidea. In the site model, no significant sites were identified in the comparison between M7 vs. M8 (Table 3). No significant loci were found in the branch model either (Table 4). The comparison in Model A vs. Model A null in the branch-site model showed statistically significant results (*p* < 0.05), identifying three amino acid sites under positive selection: amino acid residue 242 corresponds to *COX3*, residue 535 to *ND5*, and residue 138 to *ND6*. The Bayes Empirical Bayes (BEB) values for selection sites in both *COX3* and *ND6* exceeded 0.90, whereas that for *ND5* surpassed 0.95. In *COX3*, amino acid residues in the foreground branch are N/T compared to L/S/T in the background. For *ND5*, residues are S/M versus Q/Y/F, and for *ND6* they are T/S against N/S (Table 5). These findings suggest that certain amino acid sites within *Chionea* branches may be subject to positive selection.

## 4. Discussion

### 4.1. Organization of the Mitochondrial Genome

Our sequencing analysis yielded three nearly complete mitogenomes, all of which display the typical circular double-stranded structure characteristic of metazoan mitochondrial genomes. The gene arrangements of *C. tianhuashana*, *C. sphaerae* and *C. crassipes* are consistent with those observed in other Tipuloidea species, and their genomic features conform to the general characteristics of the Tipuloidea group. Mitogenome sequences from the three specimens were compared with *S. hybrida* within the subfamily Chioneinae [32], and we revealed that while most features were highly conserved, subtle differences still existed. Among the PCGs in *C. tianhuashana*, *C. sphaerae* and *C. crassipes*, 11 utilized complete stop codons, whereas two special PCGs (*COX2* and *ND5*) employed an incomplete stop codon T. Similarly, two PCGs (*ND5* and *COX2*) in *S*. *hybrida* also exhibited incomplete stop codons T, further highlighting distinctions between *Chionea* and *Symplecta*. The occurrence of partial termination codons has been widely reported in the mitogenomes of various animal groups, spanning vertebrates and invertebrates [32,72,73]. Researchers have observed that insect mitochondrial genomes exhibit a tendency for Hymenoptera species to prefer A or U as terminal nucleotides [74], while Diptera species preferentially utilize C or G as terminal nucleotides [75,76]. In the present study, the three samples terminate with T as a stop codon, which is consistent with the characteristic features of mitogenomes observed in Diptera. The canonical cloverleaf structure of tRNA molecules comprises four distinct structural domains: the acceptor stem, DHU arm, anticodon loop, and variable region. Our findings indicate that the DHU arm is absent in the trnS1 gene across all three samples, a characteristic that aligns with observations reported in other species within the superfamily Tipuloidea. The DHU arm deletion represents a conserved structural feature observed in diverse insect lineages, including *Choroterpes yixingensis* (Ephemeroptera) and *Lopaphus albopunctatus* (Phasmida) [77,78].

### 4.2. Genetic Divergence and Evolutionary Relationships

Based on the experimental results, the genetic distance between the samples *C. tianhuashana* and *C. sphaerae* collected from Jilin, China, is 5%, while the genetic distance between *C. tianhuashana* and *C. crassipes* is 5.8%, and that between *C. sphaerae* and *C. crassipes* is 6.1%. In comparison to *S.* hybrida within the same subfamily, these three samples exhibit closer phylogenetic relationships [79].

Phylogenetic reconstructions based on the 13 PCGs yielded consistent tree topologies in both Bayesian inference (BI) and maximum likelihood (ML) analyses. The adult samples of *C. tianhuashana* and *C. sphaerae*, collected from Jilin, China, cluster together to form a sister branch with *C. crassipes*. However, the monophyly of this clade remains unverifiable due to the lack of additional mitochondrial molecular data for *Chionea* species in NCBI. Our results confirm the polyphyletic status of Chioneinae, aligning with the prevailing taxonomic hypothesis first proposed by Ribeiro and widely accepted in the recent literature [29]. This study supports the monophyly of Tipulidae but does not support that of *Tipula*, contrasting with findings reported by Lukashevich et al. [30]. Furthermore, it confirms that Limoniidae is polyphyletic, as previously noted by Brodo in 1984. Additionally, this study aligns with Oosterbroek’s findings that *Nephrotoma* is also monophyletic [80]. Moreover, it supports the classification proposed by Ren et al., which posits (Pediciidae + (Limoniidae + (Tipulidae + Cylindrotomidae))) [81].

### 4.3. Selection Analysis of Three Crane Flies

Previous studies have suggested that PCGs within the mitochondrial genome may have undergone positive selection due to low-temperature influences [38,82,83]. When *Chionea* is used as a model for the foreground branch in phylogenetic analyses, evidence of positive selection becomes apparent. The prolonged cold climate during the last glacial maximum facilitated *Chionea*’s adaptation to most parts of central and southern Europe, while also extending its current southern distribution. However, similar biogeographic patterns are less evident in the New Arctic region [84]. In the branch-site model, we observed that the 242nd position of *COX3* and the 138th position of *ND6* exhibited elevated evolutionary rates, while the BEB value for the 535th position of *ND5* exceeded 0.95, indicating statistically significant results. The mitochondrial electron transport system comprises four multimeric enzyme complexes (I–IV) along with two diffusible electron shuttles (ubiquinone and cytochrome c), through which electrons released from NADH and FADH2 oxidation are transferred along these complexes before being ultimately passed to molecular oxygen [85]. Structural and functional analyses revealed that the studied mitogenome encodes key components of respiratory complexes I and IV, which are major targets of evolutionary selection. Complex I consists of seven NADH dehydrogenase subunits (*ND1*–*ND6*, *ND4L*) that collectively generate approximately 30% of cellular ATP, while complex IV incorporates the *COX3* protein as an essential catalytic core [86]. Mitochondrial oxidative phosphorylation refers to the biochemical process by which organic compounds, such as carbohydrates, lipids, and amino acids, are degraded to release energy. This process constitutes a critical biological function that supplies the essential energy required for cellular activities [87]. Complex I functions as a critical entry point for NADH (nicotinamide adenine dinucleotide) electrons into the respiratory chain, representing the initiation site of electron transport [88]. The ND series subunits constitute integral components of Complex I, and mutations in the ND5 and ND6 subunits can significantly affect mitochondrial energy production, transport efficiency, and metabolic processes [89,90]. In mouse cell mutants with altered expression of NADH dehydrogenase genes, heterogeneous mutations in the ND5 subunit result in stringent regulation of gene expression by the organism, consequently impairing respiratory function [91]. When mutations occur in the ND6 gene within this complex, the assembly of Complex I is disrupted, leading to compromised respiratory activity [92]. Moreover, Complex IV catalyzes the terminal step of electron transport in mitochondria. Its rate-limiting role during oxidative reactions serves as a key indicator of mitochondrial functionality and is closely linked to cellular energy output [93]. Within the catalytic subunit of mitochondrial complex IV, the *COX3* gene primarily acts as a transcriptional regulator and plays a crucial role in key physiological processes, including energy supply, apoptosis, metabolism, and reactive oxygen species production [94]. Mutations in the *COX3* gene, driven by environmental selection pressures, can substantially influence energy production and transfer within the mitochondrial respiratory chain.

The likelihood ratio test (LRT) for branch models is primarily employed to identify positive selection acting on a limited number of sites along a pre-specified phylogeny. However, computer simulations suggest that these tests are highly sensitive to model assumptions and may have difficulty distinguishing between the relaxation of selective constraints and true positive selection [95]. Branch-site model analysis detected just three codon positions under significant positive selection in our dataset. Previous work by Xu et al. revealed adaptive evolution in the *ND2* gene of Heptageniidae, potentially associated with cold-adaptation over extended evolutionary timescales [38]. Li et al. discovered that the *ATP8*, *COX3*, *ND2*, *ND4*, *ND4L*, *ND5*, and *ND6* genes in flying grasshoppers exhibited significant positive selection in the cold, high-altitude environment of the Qinghai–Xizang Plateau. This adaptive mechanism enhances mitochondrial gene function to better meet the energy demands of flight and the physiological challenges associated with high-altitude living [96]. The observed positive selection in *Chionea* species inhabiting cold environments may serve as an effective strategy for purging harmful mutations, thereby enhancing survival and reproductive success. While indicative of evolutionary constraints, the observed positive selection patterns require validation through broader taxonomic sampling to elucidate their mechanistic basis.

## 5. Conclusions

We assembled nearly complete mitogenomes from three *Chionea*, characterized by compositional features akin to those of insects. In the three mitogenomes of *Chionea*, the DHU arm of trnS1 is missing, a feature similar to that of most insects, and all protein-coding genes exhibited complete TAA/TAG stop codons, except for *COX2* and *ND5*, which utilized an incomplete termination codon T. Furthermore, this study supports the monophyly of Tipulidae, *Tipula*, and *Nephrotoma* and indicates that Tipulinae, Chioneinae, and Limoniidae are polyphyletic. In selection analysis, we found that three PCGs of the *Chionea* are under positive selection.

## Figures and Tables

**Table 1:** Species data utilized for phylogenetic tree construction.

Family	Subfamily	Genus	Species	Length (bp)	GenBank Accession Number
Cylindrotomidae	Cylindrotominae	*Cylindrotoma*	*Cylindrotoma* sp. ZK 2016	15,913	KT970060
Tipulidae	Ctenophorinae	*Pselliophora*	*Pselliophora bifascipennis*	15,821	NC084173
		*Tanyptera*	*Tanyptera hebeiensis*	15,888	NC053795
	Tipulinae	*Nigrotipula*	*Nigrotipula nigra*	15,400	MT483653
		*Tipula*	*Tipula cinereocincta mesacantha*	15,907	PP209203
			*Tipula varipennis*	15,772	NC086811
			*Tipula legalis*	15,625	NC086810
			*Tipula yasumatsuana*	15,735	NC086812
			*Tipula melanomera gracilispina*	14,575	MK864102
			*Tipula aestiva*	16,083	NC063751
			*Tipula fascipennis*	15,701	NC050319
			*Tipula cockerelliana*	14,453	NC030520
			*Tipula paludosa*	15,121	MT483696
			*Tipula nova*	15,668	NC057055
			*Tipula abdominalis*	14,566	JN861743
		*Nephrotoma*	*Nephrotoma flavescens*	15,269	MT628586
			*Nephrotoma quadrifaria*	16,579	MT872674
			*Nephrotoma tenuipes*	14,851	MN053900
			*Nephrotoma* sp.	17,862	OM763698
Limoniidae	Limoniinae	*Dicranomyia*	*Dicranomyia modesta*	15,311	MT628560
		*Limonia*	*Limonia phragmitidis*	15,924	NC044484
		*Dicranoptycha*	*Dicranoptycha shandongensis*	16,157	NC085228
	Limnophilinae	*Epiphragma*	*Epiphragma mediale*	14,858	NC057085
		*Elephantomyia*	*Elephantomyia inulta*	14,551	OP556661
		*Conosia*	*Conosia irrorata*	14,634	NC057072
		*Euphylidorea*	*Euphylidorea dispar*	16,069	MT410841
		*Paradelphomyia*	*Paradelphomyia* sp. ZK 2016	14,639	KT970061
		*Pseudolimnophila*	*Pseudolimnophila brunneinota*	15,985	MN398932
	Chioneinae	*Chionea*	*Chionea tianhuashana*	15,781	PV185786
			*Chionea sphaerae*	15,644	PV185785
			*Chionea crassipes*	15,260	PV185784
		*Symplecta*	*Symplecta hybrida*	15,811	NC030519
Pediciidae	Pediciinae	*Pedicia*	*Pedicia* sp. ZK 2016	14,605	KT970062
Trichoceridae		*Paracladura*	*Paracladura trichoptera*	16,143	NC016173
		*Trichocera*	*Trichocera* sp.	16,094	MW263048
			*Trichocera bimacula*	16,140	NC016169

**Table 2:** The genetic distance between *S. hybrida*, *C. tianhuashana*, *C. sphaerae*, and *C. crassipes*.

	1	2	3	4
1. *S. hybrida*				
2. *C. tianhuashana*	0.153			
3. *C. sphaerae*	0.154	0.050		
4. *C. crassipes*	0.154	0.058	0.061	

**Table 3:** Site model analysis was performed to examine adaptive evolution, with *Chionea* species assigned as the foreground branch and other Tipuloidea species as background branches.

Site model (SM)									
Model	np	Ln L	Estimates of Parameters				Model Compared	LRT P-Value	Positive Sites
M3	70	−142,014.2418	p:	0.6129	0.2908	0.0963	M0 vs. M3	0.0000	[]
			ω:	0.0035	0.0659	0.2489			
M0	66	−149,292.6487	ω0:	0.0389					Not Allowed
M2a	69	−146,919.6452	p:	0.9345	0.0233	0.0422	M1a vs. M2a	1.0000	[]
			ω:	0.0344	1.0000	1.0000			
M1a	67	−146,919.6452	p:	0.9345	0.0655				Not Allowed
			ω:	0.0344	1.0000				
M8	69	−142,234.6699	p0 = 0.9862	p = 0.3307	q = 4.7220		M7 vs. M8	0.0000	270 E 0.556, 273 N 0.798, 2830 R 0.573, 3381 K 0.619, 3497 N 0.703, 3501 I 0.590
			(p1 = 0.0138)	ω = 1.0000					
M7	67	−142,437.4345	p = 0.3481		q = 4.0661				Not Allowed
M8a	68	−141,844.7323	p0 = 0.9940	p = 0.2520	q = 4.6615		M8a vs. M8	0.0000	Not Allowed
			(p1 = 0.0060)	ω = 1.0000					

**Table 4:** Branch model analysis was conducted with *Chionea* designated as the foreground branch and the remaining Tipuloidea species serving as background branches to assess adaptive evolution.

Branch Model (BM)								
Model	np	Ln L	Estimates of Parameters			Model Compared	LRT P-Value	Omega for Foreground Branch
Two-Ratio Model 2	67	−149,287.6863	ω:	ω0 = 0.0387	ω1 = 0.05670	Model 0 vs. Two-Ratio Model 2	0.0016	ω1 = 0.0570
Model 0	66	−149,292.6488	ω = 0.0389					

**Table 5:** Positively selected sites in *Chionea* mitogenomes and amino acid divergence between foreground and background branches.

Genes	Positive Selection Sites	Amino Acids		BEB Value	Feature Key
		Foreground	Background		
COX3	1247	N/T	L/S/T	0.910	Domain
ND5	3489	S/M	Q/Y/F	0.953 *	Domain
ND6	3666	T/S	N/S	0.900	Domain

Note: * indicates BEB > 0.95.

## Data Availability

Data to support this study are available from the National Center for Biotechnology Information (https://www.ncbi.nlm.nih.gov) (accessed on 27 February 2025). The GenBank numbers are PV185784–PV185786.

## Supplementary Materials

The following supporting information can be downloaded at: https://www.mdpi.com/article/10.3390/insects16070720/s1, Table S1: The Best Model, sites, and Partition names of the seven partitions identified in the PCG123 dataset. Table S2: The characteristics of mitochondrial composition of *Chionea tianhuashuozana*. Table S3: The characteristics of mitochondrial composition of *Chionea crassipes.* Table S4: The characteristics of mitochondrial composition of *Chionea sphaerae*. Table S5: Locations of features in the mtDNA of *Chionea tianhuashana*. Table S6: Locations of features in the mtDNA of *Chionea sphaerae.* Table S7: Locations of features in the mtDNA of *Chionea crassipes*. Table S8: The RSCU of *Chionea tianhuashana*. Table S9: The RSCU of *Chionea sphaerae*. Table S10: The RSCU of *Chionea crassipes.* Figure S1: The secondary structures of all tRNAs for *Chionea tianhuashana*, *Chionea sphaerae*, and *Chionea crassipes*.
