# Supplementary material for: Comparative Mitogenomic Analysis of Three Chionea Species (Tipulomorpha: Limoniidae): Insights into Phylogenetic Relationships and Selection Pressure
Source: Insects. 2025 Jul 14;16(7):720. doi: 10.3390/insects16070720 (PMC12295488; doi:10.3390/insects16070720)
Supplement: Supplementary file 1 [file insects-16-00720-s001.zip › insects-3717172-supplementary.pdf]

Table S1. The Best Model, sites, and Partition names of the seven partitions identified in the PCG123 dataset.

Best Model	sites	Partition names
GTR+I+G	1589	*COX1*_mafft_gb_codon1, *COX3*_mafft_gb_codon1, *COX2*_mafft_gb_codon1, *Cytb*_mafft_gb_codon1, *ATP6*_mafft_gb_codon1
TVM+I+G	1704	*ATP6*_mafft_gb_codon2, *COX1*_mafft_gb_codon2, *COX2*_mafft_gb_codon2, *Cytb*_mafft_gb_codon2, nad3_mafft_gb_codon2, *COX3*_mafft_gb_codon2
TIM+I+G	1704	*COX1*_mafft_gb_codon3, *ATP6*_mafft_gb_codon3, *COX3*_mafft_gb_codon3, *COX2*_mafft_gb_codon3, nad3_mafft_gb_codon3, *Cytb*_mafft_gb_codon3
K81UF+I+G	98	*ATP8*_mafft_gb_codon2, *ATP8*_mafft_gb_codon1
TVM+G	220	nad6_mafft_gb_codon3, *ATP8*_mafft_gb_codon3
TVM+I+G	1415	nad4L_mafft_gb_codon1, nad1_mafft_gb_codon1, nad4_mafft_gb_codon1, nad5_mafft_gb_codon1
GTR+I+G	1415	nad1_mafft_gb_codon2, nad4L_mafft_gb_codon2, nad5_mafft_gb_codon2, nad4_mafft_gb_codon2
GTR+I+G	1415	nad1_mafft_gb_codon3, nad4_mafft_gb_codon3, nad5_mafft_gb_codon3, nad4L_mafft_gb_codon3
GTR+I+G	615	nad6_mafft_gb_codon1, nad3_mafft_gb_codon1, nad2_mafft_gb_codon1
TVM+I+G	500	nad6_mafft_gb_codon2, nad2_mafft_gb_codon2
K81UF+G	329	nad2_mafft_gb_codon3

Table S2. The characteristics of mitochondrial composition of *Chionea tianhuashuozana.*

Region	Strand	*Chionea tianhuashana*			
		**Length (bp)**	**AT%**	**AT Skew**	**GC Skew**
Whole genome		15781	77.2	0.032	-0.127
PCGs	+	6859	74.8	-0.129	-0.052
	-	4306	78.4	-0.263	0.233
tRNAs	+	929	76.3	0.01	0.073
	-	543	79.2	-0.033	0.239
rRNAs	-	2100	80.6	-0.039	0.289

Table S3. The characteristics of mitochondrial composition of *Chionea crassipes.*

Region	Strand	*Chionea crassipes*			
		**Length (bp)**	**AT%**	**AT Skew**	**GC Skew**
Whole genome		15260	76.7	0.033	-0.145
PCGs	+	6954	73.7	-0.116	-0.077
	-	4300	77.7	-0.261	0.225
tRNAs	+	944	76.8	0.012	0.032
	-	542	78.8	-0.04	0.304
rRNAs	-	2100	80.9	-0.036	0.282

Table S4. The characteristics of mitochondrial composition of *Chionea sphaerae.*

Region	Strand	*Chionea sphaerae*			
		**Length (bp)**	**AT%**	**AT Skew**	**GC Skew**
Whole genome		15644	77.2	0.034	-0.145
PCGs	+	6859	74.4	-0.121	-0.063
	-	4300	78.2	-0.259	0.243
tRNAs	+	945	76.2	0.006	0.031
	-	542	78.8	-0.03	0.27
rRNAs	-	2101	80.6	-0.041	0.283

Table S5. Locations of features in the mtDNA of *Chionea tianhuashana.*

Gene	From	To	Spacer(+)/ Overlap(−)	Length(bp)	Start	Stop	Strand
$tRNA^{Ile}$	1	50	0	50			H
$tRNA^{Gln}$	66	135	15	70			L
$tRNA^{Met}$	144	213	8	70			H
ND2	214	1242	0	1029	ATT	TAA	H
$tRNA^{Trp}$	1251	1322	8	72			H
$tRNA^{Cys}$	1314	1379	-9	66			L
$tRNA^{Tyr}$	1391	1458	11	68			L
COX1	1515	3014	56	1500	ATT	TAA	H
$tRNA^{Leu}$	3014	3080	-1	67			H
COX2	3085	3769	4	685	ATG	T	H
$tRNA^{Lys}$	3772	3843	2	72			H
$tRNA^{Asp}$	3852	3918	8	67			H
ATP8	3919	4083	0	165	ATT	TAA	H
ATP6	4077	4754	-7	678	ATG	TAA	H
COX3	4763	5551	8	789	ATG	TAA	H
$tRNA^{Gly}$	5551	5614	-1	64			H
ND3	5615	5968	0	354	ATG	TAA	H
$tRNA^{Ala}$	5982	6046	13	65			H
$tRNA^{Arg}$	6045	6109	-2	65			H
$tRNA^{Asn}$	6112	6178	2	67			H
$tRNA^{Ser}$	6178	6245	-1	68			H
$tRNA^{Glu}$	6245	6311	-1	67			H
$tRNA^{Phe}$	6339	6406	27	67			L
ND5	6409	8128	2	1720	ATT	T	L
$tRNA^{His}$	8137	8203	8	67			L
ND4	8203	9543	-1	1341	ATG	TAA	L
ND4L	9537	9833	-7	297	ATG	TAA	L
$tRNA^{Thr}$	9835	9901	1	67			H
$tRNA^{Pro}$	9901	9966	-1	66			L
ND6	9972	10493	5	522	ATA	TAA	H
Cytb	10493	11629	-1	1137	ATG	TAG	H
$tRNA^{Ser}$	11627	11694	-3	68			H
ND1	11711	12658	16	948	ATT	TAG	L
$tRNA^{Leu}$	12662	12727	3	66			L
16S rRNA	12728	14041	0	1314			L
$tRNA^{Val}$	14042	14114	0	73			L
12S rRNA	14115	14900	0	786			L
D-loop	14901	15781	0	881			-

Table S6. Locations of features in the mtDNA of *Chionea sphaerae.*

Gene	From	To	Spacer(+)/ Overlap(−)	Length(bp)	Start	Stop	Strand
$tRNA^{Ile}$	1	66	0	66			H
$tRNA^{Gln}$	66	135	-1	70			L
$tRNA^{Met}$	143	212	7	70			H
ND2	213	1241	0	1029	ATT	TAA	H
$tRNA^{Trp}$	1251	1321	9	71			H
$tRNA^{Cys}$	1313	1378	-9	66			L
$tRNA^{Tyr}$	1390	1457	11	68			L
COX1	1514	3013	56	1500	ATT	TAA	H
$tRNA^{Leu}$	3013	3079	-1	67			H
COX2	3084	3768	4	685	ATG	T	H
$tRNA^{Lys}$	3771	3842	2	72			H
$tRNA^{Asp}$	3853	3919	10	67			H
ATP8	3920	4084	0	165	ATT	TAA	H
ATP6	4078	4755	-7	678	ATG	TAA	H
COX3	4763	5551	7	789	ATG	TAA	H
$tRNA^{Gly}$	5551	5614	-1	64			H
ND3	5615	5968	0	354	ATG	TAA	H
$tRNA^{Ala}$	5984	6048	15	65			H
$tRNA^{Arg}$	6047	6112	-2	66			H
$tRNA^{Asn}$	6115	6181	2	67			H
$tRNA^{Ser}$	6181	6248	-1	68			H
$tRNA^{Glu}$	6248	6314	-1	67			H
$tRNA^{Phe}$	6342	6408	27	67			L
ND5	6412	8125	3	1714	ATT	T	L
$tRNA^{His}$	8140	8205	14	66			L
ND4	8205	9545	-1	1341	ATG	TAA	L
ND4L	9539	9835	-7	297	ATG	TAA	L
$tRNA^{Thr}$	9837	9903	1	67			H
$tRNA^{Pro}$	9903	9968	-1	66			L
ND6	9974	10495	5	522	ATA	TAA	H
Cytb	10495	11631	-1	1137	ATG	TAG	H
$tRNA^{Ser}$	11629	11696	-3	68			H
ND1	11713	12660	16	948	ATT	TAG	L
$tRNA^{Leu}$	12664	12729	3	66			L
16S rRNA	12730	14043	0	1314			L
$tRNA^{Val}$	14044	14116	0	73			L
12S rRNA	14117	14903	0	787			L
D-loop	14904	15664	0	741			-

Table S7. Locations of features in the mtDNA of *Chionea crassipes.*

Gene	From	To	Spacer(+)/ Overlap(−)	Length(bp)	Start	Stop	Strand
tRNA^{Ile}	1	66	0	66			H
tRNA^{Gln}	66	135	-1	70			L
tRNA^{Met}	142	211	6	70			H
ND2	212	1240	0	1029	ATT	TAA	H
tRNA^{Trp}	1249	1319	8	71			H
tRNA^{Cys}	1311	1376	-9	66			L
tRNA^{Tyr}	1388	1455	11	68			L
COX1	1457	3013	1	1557	ATT	TAA	H
tRNA^{Leu}	3013	3079	-1	67			H
COX2	3084	3768	4	723	ATG	T	H
tRNA^{Lys}	3771	3842	2	72			H
tRNA^{Asp}	3851	3917	8	67			H
ATP8	3918	4082	0	165	ATT	TAA	H
ATP6	4076	4753	-7	678	ATG	TAA	H
COX3	4762	5550	8	789	ATG	TAA	H
tRNA^{Gly}	5550	5613	-1	64			H
ND3	5614	5967	0	354	ATG	TAA	H
tRNA^{Ala}	5981	6045	13	65			H
tRNA^{Arg}	6044	6108	-2	65			H
tRNA^{Asn}	6112	6178	3	67			H
tRNA^{Ser}	6178	6245	-1	68			H
tRNA^{Glu}	6245	6311	-1	67			H
tRNA^{Phe}	6340	6406	28	67			L
ND5	6410	8123	3	1714	ATT	T	L
tRNA^{His}	8138	8203	14	66			L
ND4	8203	9543	-1	1341	ATG	TAA	L
ND4L	9537	9833	-7	297	ATG	TAA	L
tRNA^{Thr}	9835	9901	1	67			H
tRNA^{Pro}	9901	9966	-1	66			L
ND6	9972	10493	5	522	ATA	TAA	H
Cytb	10493	11629	-1	1137	ATG	TAG	H
tRNA^{Ser}	11627	11694	-3	68			H
ND1	11711	12658	16	948	ATT	TAG	L
tRNA^{Leu}	12662	12727	3	66			L
16S rRNA	12728	14042	0	1315			L
tRNA^{Val}	14043	14115	0	73			L
12S rRNA	14116	14900	0	785			L
D-loop	14901	15260	0	360			-

Table S8. The RSCU of *Chionea tianhuashana*.

Sequences used: ***Chionea tianhuashana***											
**Codon**	**Count**	**RSCU**	**Codon**	**Count**	**RSCU**	**Codon**	**Count**	**RSCU**	**Codon**	**Count**	**RSCU**
UUU(F)	332	1.91	UCU(S)	143	3.49	UAU(Y)	141	1.75	UGU(C)	41	1.95
UUC(F)	15	0.09	UCC(S)	7	0.17	UAC(Y)	20	0.25	UGC(C)	1	0.05
UUA(L)	502	5	UCA(S)	60	1.46	UAA(*)	10	1.67	UGA(W)	97	1.92
UUG(L)	23	0.23	UCG(S)	1	0.02	UAG(*)	2	0.33	UGG(W)	4	0.08
CUU(L)	36	0.36	CCU(P)	94	2.76	CAU(H)	68	1.81	CGU(R)	18	1.26
CUC(L)	1	0.01	CCC(P)	9	0.26	CAC(H)	7	0.19	CGC(R)	0	0
CUA(L)	39	0.39	CCA(P)	31	0.91	CAA(Q)	67	1.84	CGA(R)	31	2.18
CUG(L)	1	0.01	CCG(P)	2	0.06	CAG(Q)	6	0.16	CGG(R)	8	0.56
AUU(I)	345	1.95	ACU(T)	109	2.28	AAU(N)	197	1.89	AGU(S)	58	1.41
AUC(I)	9	0.05	ACC(T)	10	0.21	AAC(N)	12	0.11	AGC(S)	3	0.07
AUA(M)	214	1.83	ACA(T)	72	1.51	AAA(K)	68	1.56	AGA(S)	56	1.37
AUG(M)	20	0.17	ACG(T)	0	0	AAG(K)	19	0.44	AGG(S)	0	0
GUU(V)	112	2.26	GCU(A)	118	2.51	GAU(D)	56	1.65	GGU(G)	76	1.38
GUC(V)	7	0.14	GCC(A)	19	0.4	GAC(D)	12	0.35	GGC(G)	4	0.07
GUA(V)	77	1.56	GCA(A)	49	1.04	GAA(E)	75	1.95	GGA(G)	115	2.09
GUG(V)	2	0.04	GCG(A)	2	0.04	GAG(E)	2	0.05	GGG(G)	25	0.45
**Average# codons=3760**											

Table S9. The RSCU of *Chionea sphaerae.*

Sequences used: *Chionea sphaerae*											
**Codon**	**Count**	**RSCU**	**Codon**	**Count**	**RSCU**	**Codon**	**Count**	**RSCU**	**Codon**	**Count**	**RSCU**
UUU(F)	325	1.87	UCU(S)	142	3.43	UAU(Y)	146	1.82	UGU(C)	41	1.95
UUC(F)	22	0.13	UCC(S)	10	0.24	UAC(Y)	14	0.17	UGC(C)	1	0.05
UUA(L)	501	5.02	UCA(S)	59	1.43	UAA(*)	10	1.67	UGA(W)	95	1.88
UUG(L)	28	0.28	UCG(S)	4	0.1	UAG(*)	2	0.33	UGG(W)	6	0.12
CUU(L)	33	0.33	CCU(P)	89	2.64	CAU(H)	65	1.73	CGU(R)	18	1.26
CUC(L)	2	0.02	CCC(P)	11	0.33	CAC(H)	10	0.27	CGC(R)	1	0.07
CUA(L)	34	0.34	CCA(P)	31	0.92	CAA(Q)	70	1.94	CGA(R)	35	2.46
CUG(L)	1	0.01	CCG(P)	4	0.12	CAG(Q)	2	0.06	CGG(R)	3	0.21
AUU(I)	334	1.89	ACU(T)	105	2.22	AAU(N)	193	1.85	AGU(S)	51	1.23
AUC(I)	19	0.11	ACC(T)	13	0.28	AAC(N)	16	0.15	AGC(S)	3	0.07
AUA(M)	213	1.81	ACA(T)	70	1.48	AAA(K)	67	1.52	AGA(S)	62	1.5
AUG(M)	23	0.19	ACG(T)	1	0.02	AAG(K)	21	0.48	AGG(S)	0	0
GUU(V)	114	2.28	GCU(A)	109	2.34	GAU(D)	54	1.61	GGU(G)	65	1.18
GUC(V)	8	0.16	GCC(A)	23	0.49	GAC(D)	13	0.39	GGC(G)	4	0.07
GUA(V)	73	1.46	GCA(A)	54	1.16	GAA(E)	74	1.87	GGA(G)	125	2.27
GUG(V)	5	0.1	GCG(A)	0	0	GAG(E)	5	0.13	GGG(G)	26	0.47
**Average# codons=3758**											

Table S10. The RSCU of *Chionea crassipes.*

Sequences used: ***Chionea crassipes***											
**Codon**	**Count**	**RSCU**	**Codon**	**Count**	**RSCU**	**Codon**	**Count**	**RSCU**	**Codon**	**Count**	**RSCU**
UUU(F)	326	1.88	UCU(S)	136	3.31	UAU(Y)	128	1.58	UGU(C)	41	1.95
UUC(F)	21	0.12	UCC(S)	11	0.27	UAC(Y)	34	0.42	UGC(C)	1	0.05
UUA(L)	489	4.9	UCA(S)	61	1.48	UAA(*)	10	1.67	UGA(W)	93	1.84
UUG(L)	29	0.29	UCG(S)	4	0.1	UAG(*)	2	0.33	UGG(W)	8	0.16
CUU(L)	30	0.3	CCU(P)	86	2.55	CAU(H)	60	1.58	CGU(R)	16	1.12
CUC(L)	5	0.05	CCC(P)	11	0.33	CAC(H)	16	0.42	CGC(R)	1	0.07
CUA(L)	45	0.45	CCA(P)	37	1.1	CAA(Q)	70	1.94	CGA(R)	34	2.39
CUG(L)	1	0.01	CCG(P)	1	0.03	CAG(Q)	2	0.06	CGG(R)	6	0.42
AUU(I)	340	1.9	ACU(T)	102	2.19	AAU(N)	187	1.8	AGU(S)	59	1.43
AUC(I)	17	0.1	ACC(T)	12	0.26	AAC(N)	21	0.2	AGC(S)	8	0.19
AUA(M)	216	1.82	ACA(T)	72	1.55	AAA(K)	74	1.7	AGA(S)	50	1.22
AUG(M)	21	0.18	ACG(T)	0	0	AAG(K)	13	0.3	AGG(S)	0	0
GUU(V)	107	2.19	GCU(A)	104	2.24	GAU(D)	58	1.71	GGU(G)	63	1.14
GUC(V)	13	0.27	GCC(A)	24	0.52	GAC(D)	10	0.29	GGC(G)	10	0.18
GUA(V)	69	1.42	GCA(A)	53	1.14	GAA(E)	73	1.9	GGA(G)	104	1.88
GUG(V)	6	0.12	GCG(A)	5	0.11	GAG(E)	4	0.1	GGG(G)	44	0.8

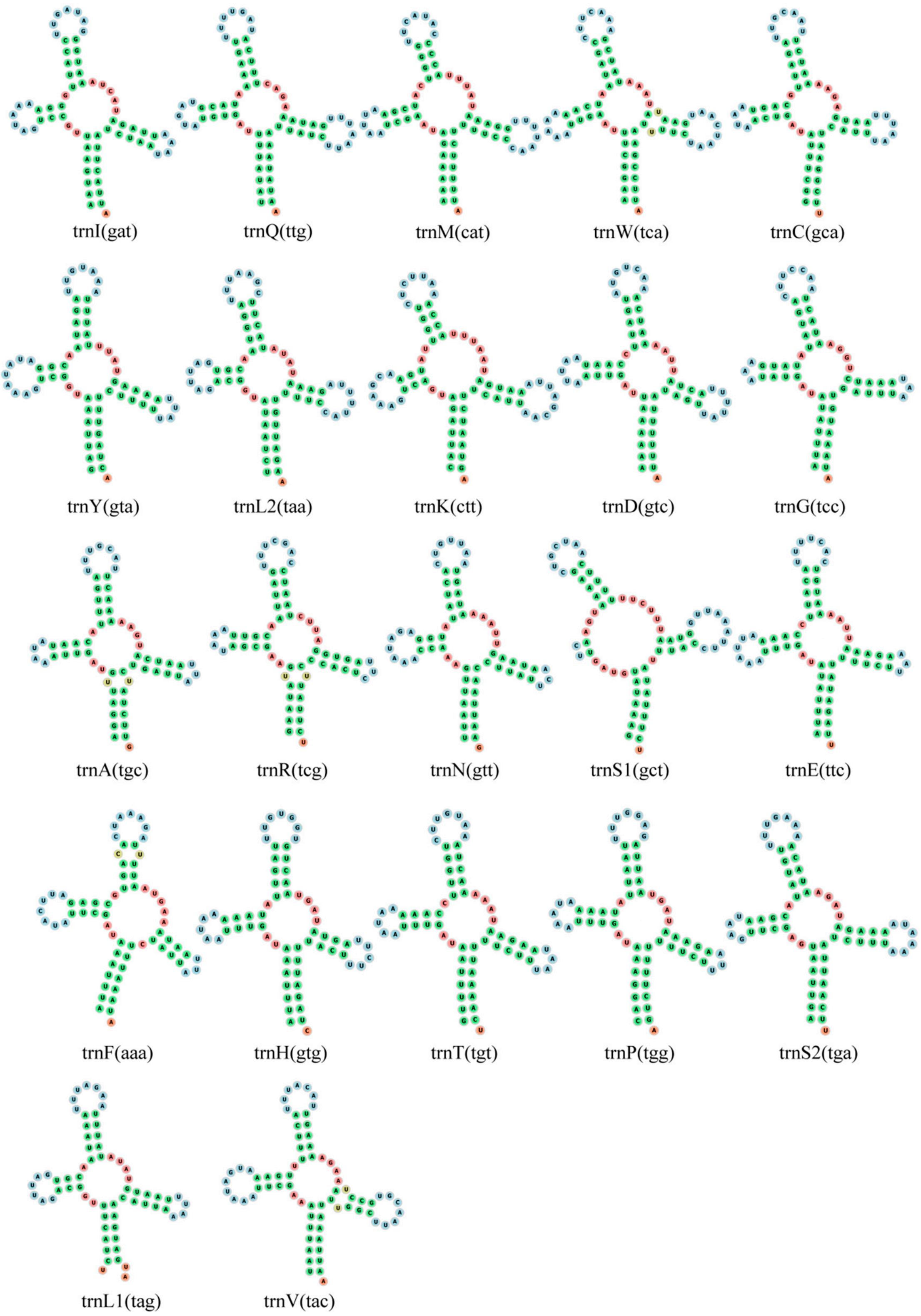

Figure S1. The secondary structures of all tRNAs for *Chionea tianhuashana*, *Chionea sphaerae*, and *Chionea crassipes*.
